# Oxidative Stress in the Blood Labyrinthine Barrier in the Macula Utricle of Meniere’s Disease Patients

**DOI:** 10.3389/fphys.2018.01068

**Published:** 2018-09-03

**Authors:** Gail Ishiyama, Jacob Wester, Ivan A. Lopez, Luis Beltran-Parrazal, Akira Ishiyama

**Affiliations:** ^1^Department of Neurology, David Geffen School of Medicine at UCLA, Los Angeles, CA, United States; ^2^Department of Head and Neck Surgery, David Geffen School of Medicine at UCLA, Los Angeles, CA, United States; ^3^Centro de Investigaciones Cerebrales, Universidad Veracruzana, Xalapa, Mexico

**Keywords:** Meniere’s disease, oxidative stress, blood labyrinthine barrier, nitrotyrosine, iNOS, pericyte migration, inflammation, vestibular

## Abstract

The blood labyrinthine barrier (BLB) is critical in the maintenance of inner ear ionic and fluid homeostasis. Recent studies using imaging and histopathology demonstrate loss of integrity of the BLB in the affected inner ear of Meniere’s disease (MD) patients. We hypothesized that oxidative stress is involved in the pathogenesis of BLB degeneration, and to date there are no studies of oxidative stress proteins in the human BLB. We investigated the ultrastructural and immunohistochemical changes of the BLB in the vestibular endorgan, the macula utricle, from patients with MD (*n* = 10), acoustic neuroma (AN) (*n* = 6) and normative autopsy specimens (*n* = 3) with no inner ear disease. Each subject had a well-documented clinical history and audiovestibular testing. Utricular maculae were studied using light and transmission electron microscopy and double labeling immunofluorescence. Vascular endothelial cells (VECs) were identified using isolectin B4 (IB4) and glucose-transporter-1 (GLUT-1). Pericytes were identified using alpha smooth muscle actin (αSMA) and phalloidin. IB4 staining of VECS was consistently seen in both AN and normative. In contrast, IB4 was nearly undetectable in all MD specimens, consistent with the significant VEC damage confirmed on transmission electron microscopy. GLUT-1 was present in MD, AN, and normative. αSMA and phalloidin were expressed consistently in the BLB pericytes in normative, AN specimen, and Meniere’s specimens. Endothelial nitric oxide synthase (eNOS), inducible nitric oxide synthase (iNOS), and nitrotyrosine were used as markers of oxidative stress. The VECs of the BLB in Meniere’s had significantly higher levels of expression of iNOS and nitrotyrosine compared with normative and AN specimen. eNOS-IF staining showed similar patterns in normative and Meniere’s specimens. Microarray-based gene expression profiling confirmed upregulation of iNOS mRNA from the macula utricle of Meniere’s patients compared with AN. Nitrotyrosine, a marker recognized as a hallmark of inflammation, especially when seen in association with an upregulation of iNOS, was detected in the epithelial and stromal cells in addition to VECs in MD. Immunohistochemical and ultrastructural degenerative changes of the VEC suggest that these cells are the primary targets of oxidative stress, and pericyte pathology including degeneration and migration, likely also plays a role in the loss of integrity of the BLB and triggering of inflammatory pathways in MD. These studies advance our scientific understanding of oxidative stress in the human inner ear BLB and otopathology.

## Introduction

In the cardiovascular, retinal, cerebrovascular and inner ear systems, homeostasis is dependent on the integrity of the selective barrier of the microvasculature. In the auditory and vestibular system, a physical and chemical barrier, the BLB tightly controls the passage of fluids, molecules, and ions to maintain a proper environment for the cellular components and a proper ionic composition of the endolymph and perilymph, while preventing the entry of deleterious substances from the vasculature to the inner ear fluid spaces (Juhn et al., 1981, 2001). Recent studies have implicated the loss of the integrity of the BLB in diverse inner ear pathologies including acoustic trauma, autoimmune inner ear disease, presbycusis, and most recently in MD (Ishiyama et al., 2017). The BLB plays a critical role in ionic transport and the maintenance of the highly differing composition of the endolymph and the perilymph. It is believed that the BLB of the inner ear is a key player in maintaining the delicate fluid balance of the inner ear. Therefore, a disease which presents nearly universally with an excess of inner ear fluid, i.e., hydrops of the endolymphatic fluid space, may be due in part to loss of BLB integrity.

Meniere’s disease is a disabling inner ear syndrome characterized by fluctuating hearing loss, recurrent episodic vertigo, aural fullness, and tinnitus with almost one-third of these patients completely disabled due to the disease and 7% of these patients suffer sudden falls, called otolithic crisis or drop attacks believed to originate from the vestibular otolithic organs, the utricle or saccule (Baloh, 2001; Ishiyama et al., 2001; Calzada et al., 2012b). Endolymphatic hydrops, a ballooning of the endolymphatic fluid space within the inner ear, is the most prominent and consistent histopathological correlate of MD, being first described in postmortem temporal bone histopathology (Hallpike and Cairns, 1938; Yamakawa, 1938). While endolymphatic hydrops is demonstrated in nearly all cases of MD (Schuknecht, 1993; Wackym, 1995) and endolymphatic hydrops can be produced in animal models (Takumida et al., 2008), researchers have been unable to replicate the spells of vertigo and hearing loss in animal models. Endolymphatic sac surgery is still practiced, however, archival human temporal bone studies and MRI imaging studies demonstrate that MD patients often have persistent hydrops on histopathology following endolymphatic sac surgery (Chung et al., 2011; Liu et al., 2016). There is growing evidence that the longitudinal flow of endolymph hypothesis of Meniere’s pathophysiology needs to be reevaluated (Ishiyama et al., 2015).

We hypothesized that the pathophysiology of MD involves dysfunction of the BLB, initially noted in gadolinium-based magnetic resonance imaging (MRI) studies that enable the visualization of endolymphatic hydrops in patients. Multiple studies have demonstrated a correlation between MD and endolymphatic hydrops, audiovestibular function, and symptom severity (Zou et al., 2005; Barath et al., 2014; Naganawa and Nakashima, 2014; Sepahdari et al., 2015). In these MRI studies, MD is associated with an increased contrast enhancement in the perilymph of the affected ear (Tagaya et al., 2011; Yamazaki et al., 2012; Barath et al., 2014; Pakdaman et al., 2016; Sepahdari et al., 2016). Intravenous gadolinium is taken up into the perilymph via perfusion through the BLB, specifically the blood perilymph barrier. In the perilymph of the ipsilateral affected inner ear, increased gadolinium uptake has been reported, and this phenomenon likely reflects loss of BLB integrity in multiple otopathologies including infection, trauma, and sudden hearing loss. In our recent study comparing MD with sudden sensorineural hearing loss, the symptomatic Meniere’s inner ear exhibits highly significantly increased contrast enhancement of the perilymph, consistent with a breakdown of the BLB, to a much higher degree than that noted in sudden sensorineural hearing loss (Pakdaman et al., 2016). Injection of intratympanic lipopolysaccharide (LPS), an inflammatory protein, is associated with an increased entry of serum fluorescein into the perilymph and causes ipsilateral increased gadolinium enhancement on MRI studies (Hirose et al., 2014; LeFloc’h et al., 2014), similar to the MRI findings in MD patients. Of note, the mechanism by which LPS induces increased vascular permeability may be through activation of iNOS in the inner ear (Takumida and Anniko, 1998a). While these findings are suggestive of increased permeability of the BLB in the affected inner ear of patients with MD, studies of the human inner ear microvasculature were needed to characterize in more detail the pathological process within the BLB in MD.

Despite more than a century of research since the original publication by Prosper Meniere in Ménière’s, 1861, the etiology of endolymphatic hydrops and its relationship with the disabling Meniere’s attacks of vertigo and hearing loss remain unknown. Our previous histopathological studies revealed neuroepithelial damage with hair cell loss, stromal edema, and subepithelial basement membrane pathology in intractable MD (McCall et al., 2009; Ishiyama et al., 2017) with questions remaining as to the cause of the neuroepithelial, stromal, and microvasculature damage. It has been shown that ischemic damage to the cochlea triggers nitric oxide production via iNOS, and is associated with increased NO metabolites within the perilymph system (Morizane et al., 2005). We proposed that oxidative stress mediated the damage to the vestibular endorgan and the BLB in MD.

Nitric oxide is likely critical in inner ear health but may also play a role in damage and oxidative damage, and is proposed to mediate neurodegenerative changes in aging (Kregel and Zhang, 2007). Nitric oxide (NO) is an endogenous neurotransmitter in the inner ear, controlling vasodilation in response to energy needs. Round window application of NO donors increases cochlear and vestibular blood flow (Arenberg et al., 1997) and intravenous infusion of NO synthase inhibitor, *N*-nitro-L-arginine-methyl ester (L-NAME) is associated with a dose-dependent decrease in cochlear vascular conductance in one study (Hoshijima et al., 2002). The constitutive nitric oxide synthase (NOS), eNOS, is ubiquitously present in the vestibular ganglion cells, nerve fibers, cytoplasm of types I and II sensory cells, nerve fibers, dark cells, transitional cells, and subepithelial microvasculature (Takumida and Anniko, 1998b; Shi and Nuttall, 2002). The presence of the inducible NOS (iNOS) is purported to be pathological and is associated with high levels of NO (Takumida and Anniko, 1998b). When NO is produced in large amounts, or in the presence of oxidative reactants, the excess NO may be associated with oxidative stress damage through the production of peroxynitrate.

To date, there are no prior studies of oxidative stress proteins in the human inner ear capillaries of the BLB, and one prior study of the NOS in the human cochlea (Popa et al., 2001). We hypothesize that MD is associated with oxidative stress markers in the BLB cellular components, inducing damage of the BLB, causing increased permeability which allows for extravasation of fluids and proteins, damaging the extracellular matrix and perivascular basement membranes.

In the present study, we investigate the ultrastructural histopathology of the BLB in MD, identified the cellular components of the BLB, presented evidence for cytochemical changes and the possible role of oxidative stress markers in BLB breakdown. Electron microscopy demonstrated endothelial damage and pericyte migration in the BLB of MD. Using immunofluorescence markers and mRNA array, we detected upregulation of iNOS and normal levels of eNOS in the macula utricle from patients diagnosed with MD. Moreover, we detected NTIF within the microvasculature of the BLB and within the epithelial and stromal cells of Meniere’s specimens. The presence of nitrotyrosine in the context of overexpression of iNOS suggests that the cellular damage is mediated by oxidative stress to the vestibular organ in MD.

## Materials and Methods

### Specimens (**Table 1**)

The Institutional Review Board (IRB) of UCLA approved this study (IRB protocol #10-001449). All methods used in this study were in accordance with NIH and IRB guidelines and regulations. Appropriate informed consent was obtained from each patient before inclusion in the study. Postmortem microdissected human vestibular endorgans were used in the present study. The temporal bone donors were part of a National Institute of Health (NIH) funded National Temporal Bone Laboratory at UCLA through the National Institute on Deafness and Other Communication Disorders (NIDCD). The medical history for each of the patients who had donated the temporal bones was maintained and preserved in a secured electronic database. A subset of specimens embedded in resin had been obtained from patients diagnosed with MD or postmortem normative from a previous study (Ishiyama et al., 2017).

**Table 1:** Specimens used in this study.

Specimen	Source	Diagnosis	Use	Figure/Table
1	S	AN	IF, TEM, LM	**Figures 2A,B**
2	S	AN	IF, TEM, LM	**Figures 4C**, **7A**
3	S	AN	IF, TEM, LM	**Figures 6A**, **8A,A1**
4	S	AN	RT2-PCR	**Table 3**
5	S	AN	RT2-PCR	**Table 3**
6	S	AN	RT2-PCR	**Table 3**
7	S	MD	IF, TEM, LM	**Figure 1B**
8	S	MD	IF, TEM, LM	**Figure 1C**
9	S	MD	IF, TEM, LM	**Figures 3A–C**, **4B**
10	S	MD	IF, TEM, LM	**Figures 4D**, **5B**
11	S	MD	IF, TEM, LM	**Figures 5C**, **6B**
12	S	MD	IF, TEM	**Figures 5D**, **7B**
13	S	MD	IF, TEM	**Figures 6C**, **8B,B1**
14	S	MD	RT2-PCR	**Table 3**
15	S	MD	RT2-PCR	**Table 3**
16	S	MD	RT2-PCR	**Table 3**
17	A	Normal	IF, TEM, LM	**Figure 1A**
18	A	Normal	IF, TEM	**Figure 4A**
19	A	Normal	IF, TEM	**Figure 5A**

*S, surgical; A, autopsy; AN, acoustic neuroma; MD, Meniere’s disease; IF, Immunofluorescence; TEM, transmission electron microscopy; LM, light microscopy; RT2-PCR reverse transcriptase, polymerase chain reaction*.

### Specimen Collection and Processing

Vestibular endorgans (macula utricle) were acquired at surgery from patients who required transmastoid labyrinthectomy for intractable vertigo and/or Tumarkin falls or from patients who required a translabyrinthine approach for acoustic neuroma resection (surgical controls). Microdissected vestibular endorgans obtained at postmortem temporal bone autopsy also served as normative controls.

### Inclusion and Exclusion Criteria

All subjects with MD had stage IV definite intractable MD with profound non-serviceable hearing loss. Subjects met the classification of definite MD in accordance with the presenting symptoms determined by the American Academy of Otolaryngology-Head and Neck Surgery (Committee on Hearing and Equilibrium, 1995) and the Classification Committee of the Barany Society (Lopez-Escamez et al., 2015). Patients who had previously undergone intratympanic gentamicin or endolymphatic shunt surgery were excluded. Unless there was a contraindication to MRI or contrast agent, all subjects had confirmation of unilateral endolymphatic hydrops in the inner ear ipsilateral to the profound hearing loss.

### Light and Transmission Electron Microscopy

Specimens were processed as described in Ishiyama et al. (2017). Briefly, for light microscopy and transmission electron microscope (TEM): Microdissected vestibular endorgans were fixed in paraformaldehyde with glutaraldehyde. Specimen collection and processing: the vestibular endorgan, the maculae utricle, obtained during transmastoid labyrinthectomy, were placed immediately in 10% buffered formalin and transported to the laboratory for post-fixation. For this study, the macula utricle was cut approximately into two halves under the dissecting microscope using micro-scissors. One half was processed for immunofluorescence and the other half was processed for light transmission electron microscopy observations.

### Tissue Processing for Immunofluorescence

Specimens were processed as described in Lopez et al. (2016). Briefly, for immunofluorescence staining one half was fixed by immersion in 10% formalin for 4 h, thereafter washed in phosphate buffered saline solution (PBS, 0.1 M, pH 7.4) for 15 min × 3 min. Tissue was then immersed in 30% sucrose diluted in PBS for 48 h. Twenty-micron thick cryostat sections were obtained using a Leica cryostat (CM1850) and mounted in Super frost plus slides (Fisher Scientific). Sections were allowed to dry at room temperature for 4 h and then stored in an ultralow refrigerator until use.

### Immunofluorescence Staining

After removal from the freezer, cryostat sections of the macula utricle were incubated at room temperature for 1 h with a blocking solution containing 1% bovine serum albumin (Faction V, Sigma), Triton X-100 (0.1%) diluted in PBS. All primary antibodies used in this study were diluted in the blocking solution as described in **Table 2**. The tissue sections were then incubated in a humidity chamber at room temperature for 16 h, washed three times for 10 min in PBS. The secondary antibody mixture was prepared using horse anti-rabbit (Alexa 488) and horse anti-mouse (Alexa 488) diluted in PBS. The antibody mixture was applied to the utricle sections for 1 h before removal. Thereafter, tissue sections were washed three times for 5 min each using PBS and then mounted with Vectashield solution containing DAPI stain to visualize cell nuclei.

**Table 2:** Antibodies and dyes used in the present study.

Antibody/dye/Source	Dilution/antibody type	SR/immunogen	Negative control	Positive control
iNOS (Abcam) (R&D)	1:500/rabbit polyclonal 1:250/mouse monoclonal	Human, mouse/GST-tagged recombinant protein corresponding to the N-terminus of mouse iNOS/NOSII	No antibody applied or pre-absorbed with antigen in the IF staining.	Human and mouse cerebellar cortex
eNOS (Chemicon) (BD Transduction)	1:200/mouse Monoclonal 1.500/Rabbit polyclonal	Human, rat, mouse/bovine eNOS	Same as above	Human cerebellar cortex
Alpha smooth muscle actin (SIGMA) (Abcam)	1:1000/mouse monoclonal 1:250/mouse monoclonal	Human, mouse, rat/N-terminal synthetic decapeptide of alpha-smooth muscle actin	Same as above	Human cerebellar cortex
Glucose transporter-1 (Abcam)	1:1000/rabbit polyclonal 1:250 rabbit monoclonal	Human, rat/synthetic peptide	Same as above	Human cerebellar cortex
Nitrotyrosine (Millipore) (Santa Cruz)	1:500/mouse monoclonal 1:100/Mouse monoclonal	Human, mouse/nitrated KHL	Same as above	Human cerebellum 98-year-old
Griffonia simplicifolia Isolectin B4 (IB-4)/Invitrogen	1:250	–	No IB4 added	Human cochlea frozen sections
Fluorescent Phalloidin/Invitrogen	1:500	–	No phalloidin added.	Mouse cochlea

*SR, species reactivity; IF, immunofluorescence.*

### Immunofluorescence Controls

For positive immunofluorescence controls, cryostat sections of the cochlea were incubated with the corresponding antibodies. Negative controls: normal macula utricle sections were incubated with all reagents except for the primary antibodies, or with the primary antibodies pre-absorbed with the corresponding antigen prepared as follow: 1 μg/1 μl of the primary antibody was mixed with 1 μg/1 μl of the antigen, and then this mixture was incubated at 37°C for 2 h. The pre-absorbed antibody was applied to the controls sections. No immunoreaction was seen in both negative controls (**Table 2**).

### Imaging

Immunofluorescence stained macula utricle sections were viewed and imaged with an Olympus BX51 fluorescent microscope (Olympus America Inc., NY, United States) equipped with an Olympus DP70 digital camera. Images were acquired using MicroSuite^TM^ Five software (Olympus America Inc.). Images were prepared using the Adobe Photoshop software program run in a Dell Precision 380 computer. Digital images were also obtained using a Leica (SP8) high resolution microscope, located in the Advanced Microscopy Laboratory and Spectroscopy (AML/S) of California Nanosystems Institute at UCLA (CNSI). Images were taken individually of each fluorescent marker, and the DAPI nuclei stain for the same tissue cross-section at 400× magnification. Images were prepared using the Adobe Photoshop software program run in a Dell Precision 380 computer.

### Transmission Electron Microscopy (TEM)

For TEM the macula utricle was processed as previously described (Ishiyama et al., 2017). In brief: half utricle was post fixed in 4% glutaraldehyde for 12 h (diluted in 0.1% buffered sodium cacodylate, pH 7.4) and then specimens were immersed in the following solutions: 2% OsO_4_ and 2% potassium ferricyanide, 0.1% thio-carbohydrazide for 1 h, 2% OsO_4_ for 30 min, uranyl acetate 1% overnight, and 0.1% lead aspartate for 30 min (with intervening 5 min × 3 min washes in double distilled water between steps). Tissue is dehydrated in ascending ethanol and embedded in resin (Epon^®^, EMS). Thin (2 microns, for light microscopy observations) and ultrathin sections (80 nm thick) were obtained using a diamond knife (Diatome) with an AO/Reichter ultracut-E microtome. Ultrathin sections were collected on single slot carbon-formvar coated copper grids (1 mm × 2 mm).

### Image Capture and Analysis

Transmission electron microscope observations and digital image capture were made using a FEI Tecnai transmission electron microscope T12 TEM −120 KV (Hillsboro, OR, United States). All sections were systematically analyzed at low (5000 magnification), and higher magnification view (22,000–25,000 magnification). Systematic analysis was performed of macula utricle sections containing blood vessels within the stroma of the maculae utricle. Sections were studied for the presence of vesicles, tight junctions in the endothelial cells, pericyte cytoplasmic organization and dendritic processes, and perivascular basement membrane alterations (i.e., thickening and disruption), and overall organization of the vestibular sensory epithelia.

### Quantification of Immunofluorescence

Quantification of immunofluorescence signal was obtained and comparisons were made between MD and acoustic neuroma and MD and normative postmortem microdissected utricle stained sections. Quantitative analysis was made as described from the study (Ishiyama et al., 2010; Balaker et al., 2013), using *ImageJ* software^1^ (version 1.50g). Individual images captured were opened using the *ImageJ* program and converted to gray scale (image > type 8 bit). The threshold was set (image > adjust > threshold) and adjusted to the same values for all images. Background immunofluorescence signal was measured in a small area located apart from the specific antibody staining and subtracted to show only specific staining (Process > Set Background). The image was converted to black and white, and the immunofluorescence area (blood vessel underneath the stroma) was selected using the selection tool. To determine the immunofluorescence area within the region of interest the command (analyze > analyze particles was selected), and the “mask tool” was selected. The resulting information obtained was the immunofluorescence area fraction, which is the proportion of the region of interest that was immunofluorescent. Values obtained in the normative and acoustic neuroma utricle (pixels) were considered 100%. This value was compared against Meniere’s utricle stained sections.

### Statistical Analysis

We tested whether the immunofluorescence area in the Meniere’s utricle was statistically different from the normative and acoustic neuroma. For each specimen, mean values of the immunofluorescence area was averaged and subjected to one-way repeated measurements analysis of (ANOVA). Comparison were made between the Meniere’s utricle sections and acoustic neuroma sections, and between Meniere’s utricle sections and normative (autopsy) sections, for each immunofluorescence marker. A *p*-value ≤ 0.05 indicates a statistically significant different change in the immunofluorescence area. The Sigma Stat 3.1 software program (Jandel Scientific, San Rafael, CA, United States) was used for statistical analysis.

### Gene Expression Array

We used a custom real time RT profiles PCR array^TM^ (RT^2^ Profiler^TM^ PCR Array Human Oxidative Stress cat # PAHS-065Z, SABiosciences), to simultaneously evaluate the expression of 84 genes associated with oxidative stress in the macula utricle from Meniere’s patients and compared their expression with acoustic neuroma group and perform a by qPCR and performed a 2^−(ΔΔCT)^ analysis. Fold regulation was normalized [baseline expression is represented by a 2^−(ΔΔCT)^ = 1], we considered that a gene significantly increased its expression only if it was larger than five times the baseline expression, i.e., 2^−(ΔΔCT)^ ≥ 5.

For this purpose, utricles were received into RNAlater (Ambion, Texas) and taken immediately to the laboratory and keep overnight in the refrigerator (4°C). Thereafter the specimens were placed at −80°C until mRNA was extracted. See **Table 1**: A pool of three utricles was used for each mRNA assay per group (Meniere’s and acoustic neuroma). Total RNA was extracted using TRIzol reagent (Invitrogen) and amplified using a single-color real-time polymerase chain reaction (PCR) system (Stratagene Mx3000P) as described before (Beltran-Parrazal et al., 2010; Ishiyama et al., 2010). Given that the amount of RNA obtained is minimal, RT^2^ Nano Pre-AMP technology was required to synthesize cDNA from nanogram amounts of RNA samples (1–100 ng). This technique utilizes multiplex PCR-based pre-amplification to provide amplification of gene-specific cDNA target templates with minimal bias.

RT-Polymerase Chain Reaction: Pre-amplified cDNA and RT^2^ qPCR SYBR Green Master Mix was then loaded onto 96-well RT^2^ Profiler PCR Arrays (SABiosciences) specific for gene detection of human oxidative stress. The criteria to identify statistically significant changes from control values was a *p*-value less than 0.05 and a mean difference equal to or greater than twofold. Comparison was made between utricles from Meniere’s specimens and acoustic neuroma specimens.

## Results

### Vestibular Endorgans in Meniere’s Disease and Normal

At the light microscopy level, there were consistent histopathological changes noted in the microvasculature, neuroepithelium, and stroma when comparing the Meniere’s specimen with postmortem normative. The endorgans from Meniere’s patients exhibited edematous changes of the stroma and the neuroepithelium, and subepithelial basement membrane thickening.

#### Normative

**FIGURE 1:**
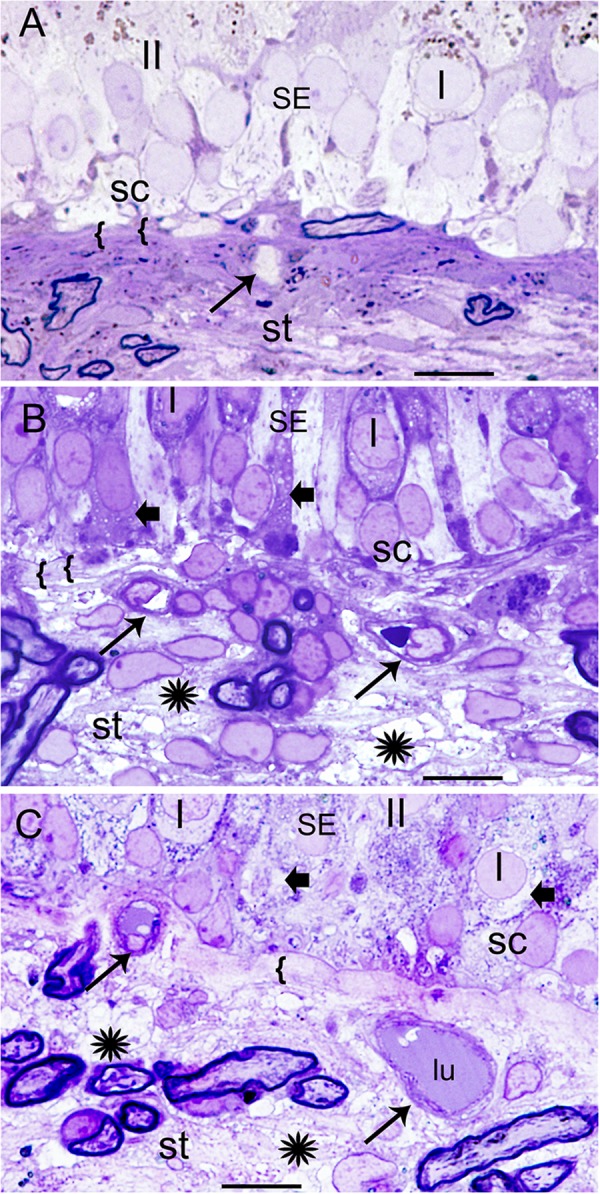
Histological changes in the normative and Meniere’s disease macula utricle. **(A)** Normal postmortem autopsy in a 70-year-old male with no vestibular dysfunction, { { points to the normal, thin, uniform basement membrane underneath the sensory epithelia. I, Type I hair cells; II, type II hair cells; st, stroma; sc, supporting cells. Arrow points to a capillary in the stroma. **(B)** Meniere’s disease utricle from a patient diagnosed with intractable vertigo (65-year-old male, surgical). There is edema within the stroma, and a thickened basement membrane. **(C)** Meniere’s disease with delayed hydrops, vertigo spells and Tumarkin falls. Asterisks show signs of edema in the stroma in **(B,C)**; SE, sensory epithelia. Arrowheads in the sensory epithelia indicated intracellular edema. Arrowhead point to dilated microvasculature in the stroma in **(B,C)**. { {: in **(B,C)** pronounced thickening of the basement membrane. One-micron thick plastic sections counterstained with toluidine blue. Bar is 10 microns.

**Figure 1A** demonstrates the normative histology from a postmortem acquired vestibular utricular macula from a 70-year-old male with no auditory or vestibular pathology. The overlying neuroepithelium exhibits normal cytoarchitecture of types I and II hair cells, with supporting cells beneath, with only very mild prominence of the subepithelial basement membrane, known to occur with aging, for example, in retinal basement membranes (Nagata et al., 1986). The underlying subepithelial basement membrane is smooth, uniform, orderly, and without duplication or thickening, barely perceptible in the normative vestibular endorgan. The stromal vasculature exhibits regular arrangement of the capillaries without dilatation or necrosis.

#### Meniere’s Disease

**Figures 1B,C** shows the vestibular sensory epithelia from two patients with MD. Supporting cells showed vacuoles, and the subepithelial basement membrane is thickened, disorganized. There are edematous changes throughout the vestibular stroma particularly increased in proximity to the subepithelial basement membrane, and in areas rich with perivascular stromal capillaries. The subepithelial basement membrane separating the hair cells (hc) and supporting cells (sc) from the stroma was thickened, rarefied, and ranged from mildly (as shown) to severely disorganized. The stroma exhibited edema and vacuolization. There was edema and vesicles in the supporting cells and hair cells as well. The cross-section diameter of the blood vessels underneath the stroma of the normal human utricle generally ranges from 8 to 12 μm. Blood vessels in this Meniere’s specimen generally ranged from 14 to 16 μm.

### Microvasculature in the Stroma of a Normal and Meniere’s Disease Utricle (Transmission Electron Microscopic)

#### Acoustic Neuroma

**FIGURE 2:**
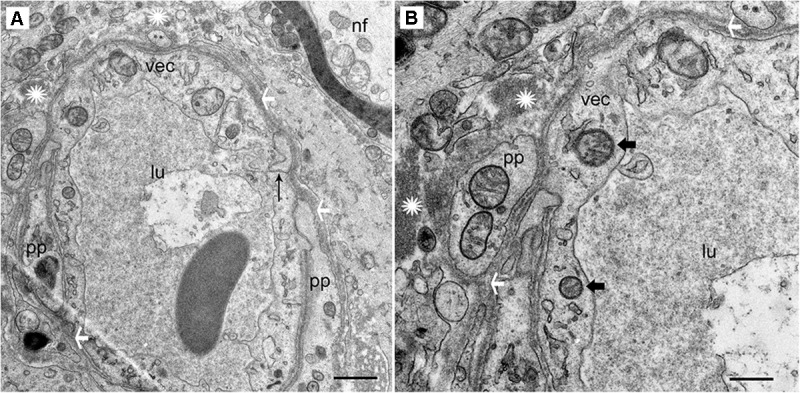
Transmission electron photomicrographs of blood vessels in normal macula utricle (acoustic neuroma). **(A)** Cross section of a capillary (low magnification view) located in the stroma beneath the macula utricle sensory epithelia. **(B)** On higher magnification, the VECs (vec) are smooth with few vesicles, the lumen (lu) is without fenestrations, and the tight junctions (thin black arrow) appear to be unaltered. pp, pericyte processes are normal without migration or thinning, ^∗^ asterisk shows the normal compact, uniform perivascular basement membrane, white arrowheads also point to the dense perivascular membrane; nf, normal nerve fiber axoplasm. Thick arrowhead: points to normal mitochondria. Magnification bar in **(A)** is 2 μm; **(B)** is 500 nm.

The stromal capillary BLB in acoustic neuroma is relatively normal (**Figures 2A,B**). The VECs form a continuous, smooth, capillary lining with few vesicles, the lumen is without fenestrations, and the tight junctions appear to be unaltered. The VECs contain normal subcellular organelles, with several intact mitochondria noted. A pericyte process is making continuous, smooth contact with the VEC without any detachment or migration. The basement membrane is thin and uniform. Because normative inner ear surgical specimens are available only rarely, acoustic neuroma is used as normative.

#### Meniere’s Disease

In contrast, the BLB capillaries underneath the utricle sensory epithelia from a patient with Tumarkin falls (proposed to originate from the vestibulospinal reflex in the utricle or saccule) exhibits moderately severe BLB histopathology. In this case of active MD with Tumarkin falls, within the utricle there is moderately severe with dilation of the vasculature, edema formation in the extracellular matrix, and basement membrane thickening.

**FIGURE 3:**
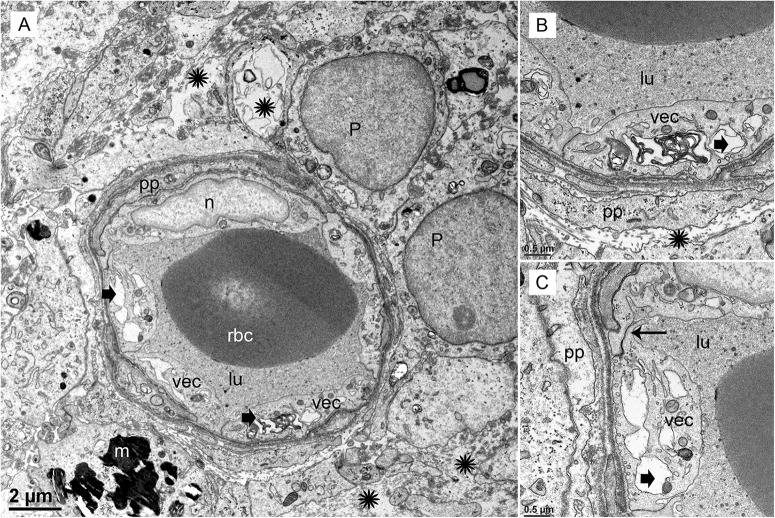
Transmission electron photomicrographs of blood vessels in Meniere’s disease macula utricle. **(A)** Low power, and **(B,C)** high power magnification shows a blood vessel with marked alterations and atrophy. The VECs (vec) are swollen, and the cytoplasm vacuolated (thick black arrowheads). The perivascular basement membrane is irregular, and there are edematous changes within the extracellular matrix (black asterisks). Multiple vesicles on the abluminal face of VEC with opening onto the perivascular basement membrane can be seen. p, pericytes; pp, pericyte process; m, melanin pigment in a putative perivascular macrophage. rbc, red blood cells; lu, lumen, thin arrow in **(C)** shows a normal tight junction between to VECs. Magnification bar in **(A)** is 2μm; **(B,C)** are 0.5 μm.

Under TEM, pericyte migration and severe edematous changes are noted in the capillaries of the BLB in the utricle (**Figures 3A–C**). The VECs are swollen, the perivascular basal lamina is irregular, and there are edematous changes within the extracellular matrix. There is not ultrastructural evidence for disruption of the tight junctions. Higher magnification view shows that the cytoplasm of VECs is vacuolated. Multiple vesicles on the abluminal face opening onto the basal lamina can be seen. Within the VEC, there are fragments of degraded subcellular organelles. There are two pericytes with a pattern of migrating, with loss of the encircling continuous projections covering the capillary. The pericyte in the lower right side has apparent space between the process and the endothelial cell, indicating detachment. There is excessive stromal edema with large vacuoles and debris within the VEC.

### Cytological Identification of VECs and Pericytes and Alterations in Cell-Specific Markers; Damaged Endothelial Cells in Meniere’s Disease Exhibited Nearly Absent Isolectin B4 (IB4) Expression

#### Isolectin IB4 Expression in Endothelial Cells Is Nearly Absent in Meniere’s Disease, While GLUT-1 Expression Appears to Be Unchanged

**FIGURE 4:**
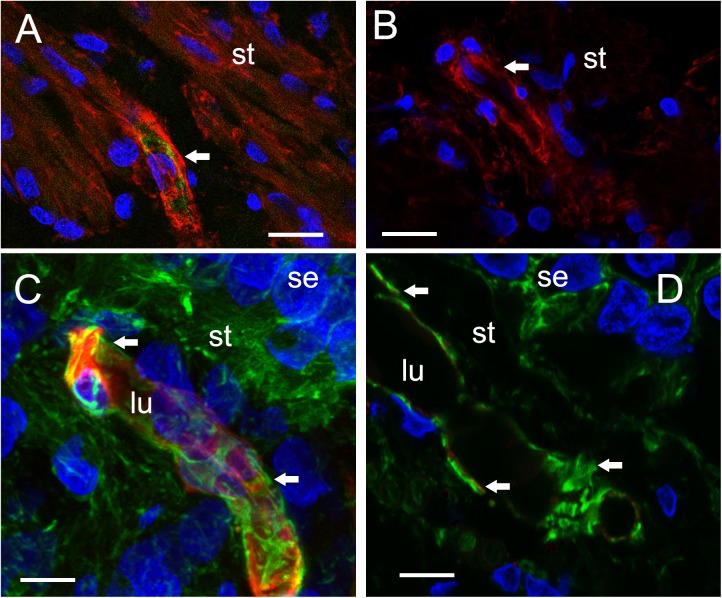
Identification of VECs and pericytes main cellular components of the BLB. **(A)** *Normative* vs. **(B)** *Meniere’s disease*: Laser confocal fluorescence micrographs showing the identification of VECs with IB4-IF, and pericytes with actin (phalloidin-F) staining in the vasculature located in the stroma of macula utricle from normal (autopsy) and Meniere’s disease specimens. **(A)** Micrograph shows autopsy specimen (62-year-old-female). The pericyte processes (red phalloidin) are surrounding the capillary with VECs expressing IB4 (green IB4). **(B)** Micrograph shows a Meniere’s disease specimen high magnification view. Arrowheads point to actin staining (red), IB4-IF in green. In the Meniere’s specimen, there is a lack of expression of IB4. In **(A,B)** and, phalloidin was labeled with Alexa 594 (red) and IB4-IF was labeled with Alexa 488 (green). **(C)** *Normative acoustic neuroma* vs. **(D)** *Meniere’s disease*: **(C)** Capillary from a normative acoustic neuroma (58 year-old-male), and **(D)** capillary from a Meniere’s specimens (59 years old female). In **(C,D)**, phalloidin was labeled with Alexa 488 (green) and IB4 with Alexa 594 (red). Both **(C,D)** are laser confocal stacked images to reconstruct a 3-dimensional view. The pericytes processes can be visualized as wrapping the capillary in fingerlike processes (green, white arrowheads). VECs are also well identified by IB4 expression (red). There is a lack of IB4 staining in the blood vessel of the Meniere’s specimens. DAPI stain cell nuclei (blue). St, stroma; se, sensory epithelia; lu, blood vessel luminal portion. Magnification bar in **(A,B)** is 10 microns. In **(C,D)** is 5 microns.

The VECs of the BLB are highly specialized, similar to the endothelial cells of the BBB. These cells form a single layer, tightly packed, with a greater density of mitochondria than non-neural endothelial cells. Isolectin IB4 can be used as a vascular stain for the study of VECs demonstrated in a colocalization study using IB4 (Benton et al., 2008) and a known pan-endothelial monoclonal antibody vasculature marker, RECA-1 (Duijvestijn et al., 1992; Ernst and Christie, 2006). In the specimens from MD, there was no discernable IB4 expression in the microvasculature compared with the normative postmortem control (**Figures 4A** vs. **4B**: note lack of green IB4-IF in **Figure 4B**). This likely reflects the severe degeneration of the endothelial cell. While isolectin IB4 identifies capillaries, small and medium sized arteries can be identified with von Willebrand A domain-related protein (Duong et al., 2011). Similarly, in surgical normative acoustic neuroma, the IB4-IF was noted in red, with stellate, fingerlike pericyte processes in green (**Figure 4C**) but the Meniere’s specimen lacked IB4 fluorescence (**Figure 4D**).

### Pericyte Expression of α Smooth Muscle Actin (SMA) and Phalloidin Appears to Be Unaltered in Meniere’s Disease Compared With Normative; In Normative, Pericytes Are Noted to Wrap in Fingerlike Projections Around the Capillary of the BLB and in Meniere’s Pericytes Exhibit Degradation/Migration

There is no obvious alteration in expression of smooth muscle actin or phalloidin in MD microvasculature of the vestibular stroma. There is a hint of increased expression of SMA [but not significant alteration of expression (see **Table 3**)] which may occur in the setting of pericyte detachment and migration (**Figure 4**). **Figures 4A,B** demonstrate the expression of SMA within the pericyte is similar comparing normative surgical control (acoustic neuroma) with MD. Phalloidin staining using laser confocal microscopy allowed the full visualization of the pericyte processes: in the normative, there was the common pattern of pericytes encircling the capillary with projections that cover the capillary with fingerlike projections (**Figure 4C**).

**Table 3:** Analysis of quantitative immunofluorescence.

Marker	MD vs. AN	% fold change from AN	MD vs. normative	% fold change from normative
Isolectin IB4	*p* ≤ 0.05	^∗∗^95	*p* ≤ 0.05	^∗∗^90
Actin	*p* ≥ 0.05	^∗^5	*p* ≥ 0.05	^∗^7
GLUT-1	*p* ≥ 0.05	^∗^2	*p* ≥ 0.05	^∗∗^5
iNOS	*p* ≤ 0.05	^∗^90	*p* ≤ 0.05	^∗^90
αSMA	*p* ≥ 0.05	^∗^6	*p* ≥ 0.05	^∗^4
eNOS	*p* ≥ 0.05	^∗^8	*p* ≥ 0.05	^∗^3
Nitrotyrosine	*p* ≤ 0.05	^∗^90	*p* ≤ 0.05	^∗^95

*A p-value p ≤ 0.05 represents a statistically significant difference between immunofluorescence mean area, p ≥ 0.05 represent no statistically significant difference. ^∗^ Indicates % of increase or ^∗∗^ % of decrease in immunofluorescence. MD, Meniere’s disease; AN, acoustic neuroma.*

### The VECs in the Human BLB Express Glucose Transporter – 1 (GLUT-1) Similarly to the Human BBB (**Figure 5A** Normative and **Figures 5B–D**: Meniere’s)

**FIGURE 5:**
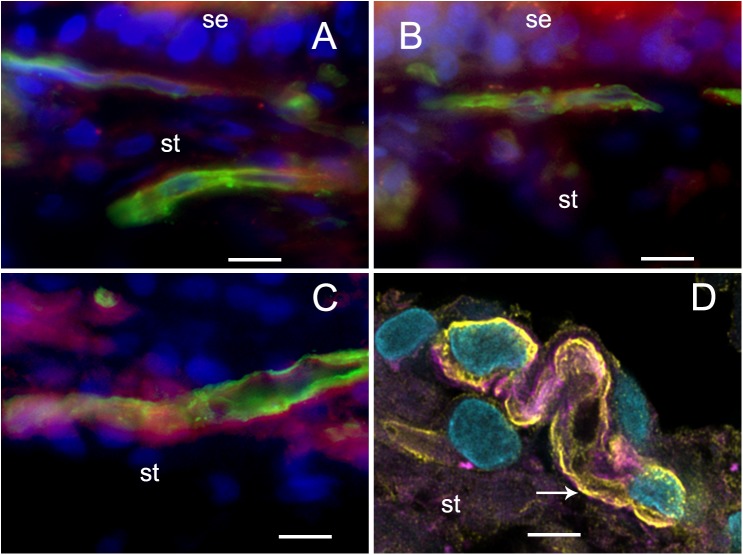
Glucose transporter-IF (VECs, green) and Phalloidin actin (pericytes, red) **(A)**, Normative, **(B–D)**. **(A–C)** Fluorescent microscopy images. Meniere’s specimens. GLUT-1-IF was consistently present in all the specimens examined (normative and Meniere’s specimen). **(D)** Shows a laser confocal image yellow color (white arrow) shows GLUT1 in VECs, pink color shows actin (phalloidin) in pericytes, and clear blue shows cell nuclei (DAPI). St, stroma; se, sensory epithelia. Magnification bar **(A–C)** is 15 microns; in **(D)** is 5 microns.

Because neurons and sensory cells require a continuous supply of energy, and use primarily glucose as fuel, the BBB endothelial cells universally express high levels of GLUT-1 in the luminal and abluminal membrane of the endothelium (Farrell and Pardridge, 1991). These integral membrane proteins help the glucose be transported down the gradient from the circulation to the endothelial cell, and then to the interstitium and then to neurons or sensory cells. GLUT-1 can be recruited when needed for extra energy to be expressed in the plasma membrane (Farrell and Pardridge, 1991). The BLB endothelial cell exhibited high levels of GLUT-1, similarly localized as previous studies noted in the human BBB. There was no difference in expression of GLUT-1 in MD from normative surgical control, acoustic neuroma.

### Inducible Nitric Oxide Synthase (iNOS) Is Upregulated and There Is an Increase Expression of Nitrotyrosine in the Capillaries of the BLB, the Stroma, and the Neuroepithelium in Meniere’s Disease

**FIGURE 6:**
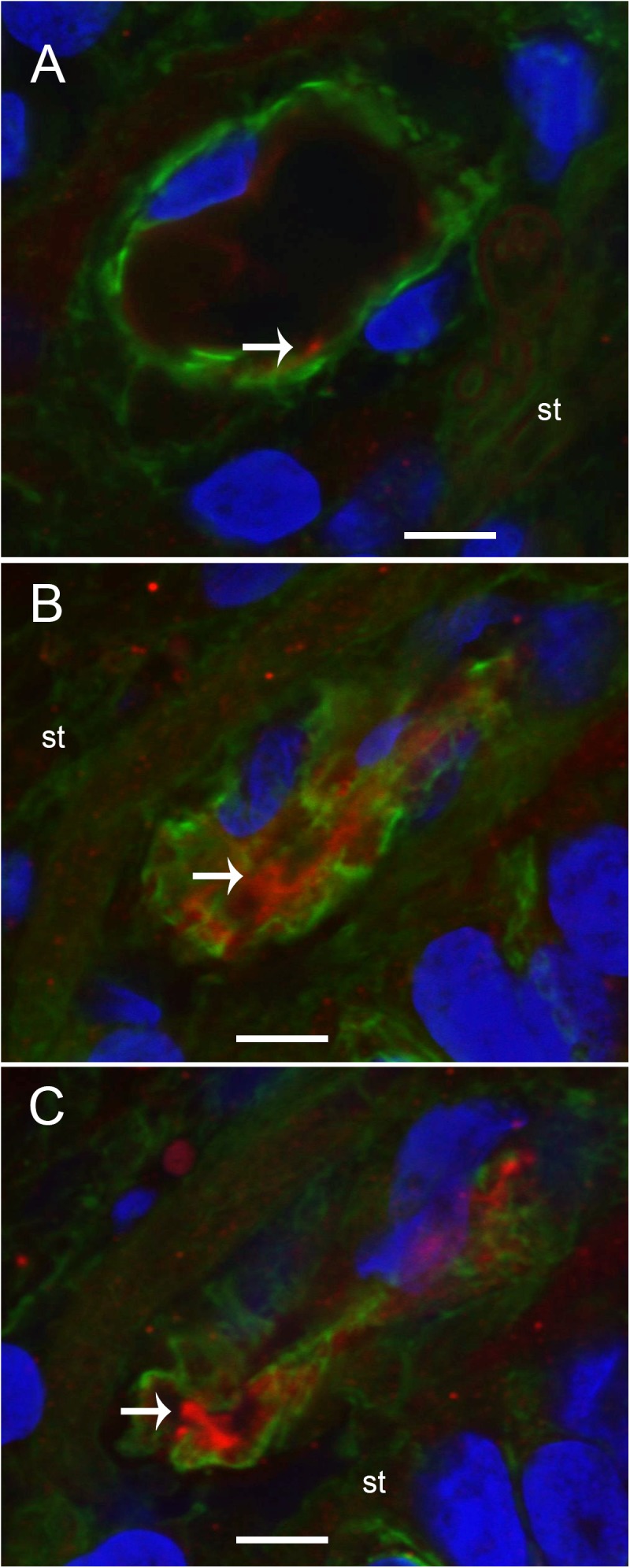
Laser confocal photomicrographs showing the presence of inducible nitric oxide synthase-IF (iNOS-IF, red) and αSMA-IF (green) in the normal and Meniere’s macula utricle vasculature. **(A)** Blood vessels in an acoustic neuroma specimen, **(B,C)** blood vessels from Meniere’s disease utricle stroma (st). iNOS-IF was present in all Meniere’s disease specimens examined (white arrow) while normative and acoustic neuroma showed minimum small area of iNOS-IF. Magnification bar in **(A–C)** are 5 microns.

**FIGURE 7:**
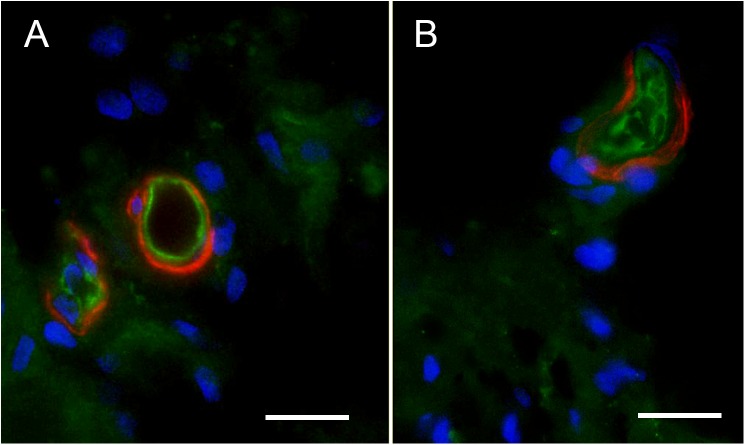
Endothelial nitric oxide synthase (eNOS)-IF in the surgical normative (acoustic neuroma) and Meniere’s macula utricle microvasculature stroma. **(A)** Shows eNOS-IF in the VECs (green), and αSMA-IF in pericytes (red) in acoustic neuroma specimen, **(B)** shows eNOS-IF in the blood vessels VECs (green) and αSMA-IF in Meniere’s disease. Both specimens showed eNOS-IF in a similar intensity. Magnification bar in **(A,B)** are 10 microns.

We conducted immunohistochemical localization of iNOS and eNOS in the specimens from MD and compared with surgical normative, acoustic neuroma. iNOS was expressed in all vestibular inner ear specimens with severe intractable MD, but was not present in any of the acoustic neuroma specimens. The VECs in Meniere’s specimens showed significantly higher expression of iNOS than the acoustic neuroma utricle. (**Figures 6A–C**). In contrast, eNOS, the constitutive NOS, appeared to be equally expressed within the VECs of the BLB in both surgical normative and MD (**Figures 7A,B**).

### Nitrotyrosine Immunofluorescence (NT-IF) Is Noted in the Capillaries of the BLB, the Stroma, and the Neuroepithelium of Meniere’s Specimens but Not in Normative Surgical Controls

**FIGURE 8:**
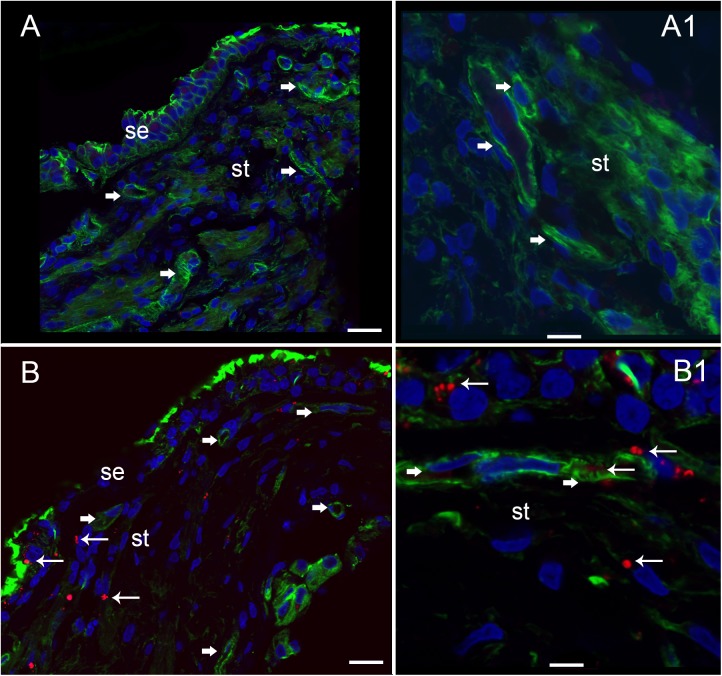
Nitrotyrosine-immunofluorescence (NT-IF, red color) and actin (Actin-IF, green color) in the utricle of normative (acoustic neuroma) and Meniere’s disease (all images obtained using laser confocal microscope). **(A)** Low magnification view showing almost complete absence of NT-IF in the normal utricle stroma (st) and epithelial cells (se), actin delineate a capillary (Phalloidin green color, arrowhead) **(A1)** high magnification showing actin (phalloidin in green) around the vasculature (arrowheads). **(B)** In contrast NT-IF (red, thin arrows), was present in the sensory epithelial (se) cells, stroma and VECs, **(B1)** is a high magnification view showing NT-IF around and inside a capillary (thin arrows); st, stroma. Magnification bar in **(A,B)** are 25 microns; **(A1,B1)** are 10 microns.

Nitrotyrosine-IF was not detected in the vestibular utricular maculae from surgical normative specimen (acoustic neuroma) as noted in **Figure 8A**. In striking contrast, NT-IF was detected throughout the utricular maculae in all MD specimens examined. In the macula utricle from Meniere’s patients, NT-IF was found within the cells of the sensory epithelia and stroma, as well as within the capillaries of the BLB within the stroma (**Figure 8B**). Nitrotyrosine is a post-translational modification to proteins, and the addition of a ^−^NO_2_ group to the exposed protein tyrosine is a stable transformation that does not occur at random. Nitrotyrosine can be formed by the reactive species, peroxynitrite, and is generally believed to indicate oxidative stress (Peluffo and Radi, 2007). There are other nitrating agents, however, in the setting of co-expression of iNOS, which is normally not expressed, it is believed that nitrated proteins, nitrotyrosine, are formed due to high NO production in the setting of oxidative stress (Beckman and Koppenol, 1996). The resultant oxidative stress, in the form of NO and nitrotyrosine, may play a critical role in the loss of integrity of the BLB, and may lead to the formation of stromal and neuroepithelial degenerative changes and vacuolization of the stroma and of cellular structures.

### Quantitative Analysis of Immunofluorescence

Comparison of immunofluorescence of the different markers between the specimens from MD vs. normative revealed the following significant changes (**Table 3**): isolectin IB4 exhibited a statistically significant decreased expression in the endothelial cells of the BLB of Meniere’s specimens, compared with the expression in acoustic neuroma (surgical normative) and normative (autopsy). Actin, GLUT-1, αSMA, and eNOS demonstrated unaltered expression levels among the three types of specimens. In contrast both iNOS and nitrotyrosine showed statistically significant increased levels of expression in the vestibular BLB of Meniere’s specimens.

### Microarray-Based Gene Expression Profiling Demonstrates Significant Increase in iNOS mRNA in Meniere’s Utricles

Meniere’s disease utricles showed significantly iNOS mRNA increase (i.e., larger than five times) with respect to acoustic neuroma utricles (more than five times increase) (**Table 4**). Additional mRNAs in the Meniere’s specimens showed significant changes: Albumin, Dual specificity phosphatase 1, Polynucleotide kinase 3′-phosphatase, Peroxidasin, Selenoprotein P, Serine/threonine kinase 25 (**Table 4**).

**Table 4:** Gene expression analysis by qPCR of genes related to oxidative stress in Meniere Disease (MD) vs. Acoustic neuroma (AN).

Name	ID GeneBank	2^−(ΔΔCT)^
**Albumin ↓**	NM_000477.3	**-5.1**
Arachidonate 12-lipoxygenase.	NM_001159.3	-2.2
Catalase	NM_001752.2	-4.5
24-dehydrocholesterol reductase	NM_014762.3	4.1
**Dual specificity phosphatase 1 ↓**	NM_004417.2	**-5.2**
Forkhead box M1	NM_021953.2	-2.2
Glutathione peroxidase 3	NM_002084.3	-2.1
Glutathione *S*-transferase zeta 1	NM_001513.2	-2.3
Lactoperoxidase	NM_006151.1	-2
Metallothionein 3	NM_005954.2	-1.8
PDZ and LIM domain 1	NM_020992.2	-2.5
**Polynucleotide kinase 3′-phosphatase ↓**	NM_007254.2	**-7.5**
Prion protein	NM_183079.2	1.8
**Peroxidasin ↓**	NM_144651.4	-8.5
**Selenoprotein P ↓**	NM_005410.2	-5.1
**Serine/threonine kinase 25 ↓**	NM_006374.3	-5
**Nitric oxide synthase 2**	NM_000625.3	**13.43**
Oxidation resistance 1	NM_001198532.1	-2.9

*Upregulated genes (green), downregulated (red). Arrows represent statistically significant increase or decrease in fold regulation. Bolded values indicate a statistically significant increase or decrease (more that 5 times) of mRNA expression.*

## Discussion

### Vestibular Endorgans Exhibit Damage in the Vasculature, Stroma, and Neuroepithelium

The vestibular sensory endorgan in MD exhibits stromal vacuolization, neuroepithelial damage to the sensory hair cells and supporting cells, and a thickened, irregular subepithelial basement membrane. The edematous changes throughout the vestibular stroma are increased in proximity to the subepithelial basement membrane, and in areas rich with stromal capillaries. There was edema and vesicles within the supporting cells and hair cells, and the subepithelial basement membrane exhibited thickening and damage. The cross-sectional diameter of the blood vessels underneath the stroma of the normal human utricle generally ranges from 8 to 12 microns and the vessels in Meniere’s specimens generally range from 14 to 16 microns.

### The Blood Labyrinth Barrier

The BLB, specifically, the blood perilymph barrier, localizes to the microvasculature of the vestibular stroma, spiral ligament, spiral limbus, and modiolus (Suzuki and Kaga, 1999). The ultrastructure in the capillaries of the vestibular stroma in human demonstrates similarities with the BLB in animal models and is also similar to the cytoarchitecture of the blood–brain barrier (BBB) (Reese and Karnovsky, 1967; Nag, 2003). In the present study, we confirm that the integrity of the normative human BLB is maintained by VECs forming a continuous, non-fenestrated lining with few vesicles, sparse transcytosis, tight junctions appearing normal, smooth-lined basement membrane with pericytes intermingled and entirely encompassed within the basement membrane (Ishiyama et al., 2017). The BLB is similarly structured in the guinea pig spiral limbus and modiolus (Jahnke, 1980) and mouse strial BLB (Shi et al., 2008; Wu et al., 2014).

In the present study, we did visualize a putative melanin containing perivascular macrophage surrounding the capillary of the vestibular BLB in Meniere’s specimen. The rarity with which we see the perivascular macrophage may indicate species-specific differences or differences due to pathological states. Pericytes are known to exhibit pluripotent activity, and the migrating pericyte can be seen in pathological states and may exhibit macrophage like characteristics (Dore-Duffy, 2008).

### Structural and Cytochemical Changes in the BLB in Meniere’s Disease

#### TEM of Vascular Endothelial Cell (VEC) and Pericytes

In the vestibular endorgans from MD, the VECs exhibit increased vesicular formation facing the abluminal side, edema and vacuolization of the perivascular basement membrane. The tight junctions did not exhibit changes that could be discerned at the ultrastructural level. However, it has been noted that without using tracers, the opening of the tight junctions may not be visualized (Hirano et al., 1994) and tight junctions in the BBB can demonstrate rapid reversible opening (Rapoport et al., 1972). However, we cannot rule out tight junction pathology with structural imaging, and physiological studies in patients cannot be conducted.

There was VEC degeneration with loss of organelles; and confirming the cellular damage, the marker isolectin-IB4 was nearly universally absent in the VEC of the MD specimens but was present in both acoustic neuroma and postmortem normative. It is informative to evaluate these findings in light of other systems, such as the BBB in normative and pathology. In normative conditions, the BBB endothelial cell from cerebral cortex contain only 5 caveolae per micron ^2^ likely intended to limit transcellular traffic in steady state. With hypertensive encephalopathy, there is an increase in vesicular transport in the endothelium, and there are structural changes in the tight junctions. It is believed that these structural changes occur late and are preceded by endothelial cell breakdown (Nag et al., 2011). Similar patterns of endothelial cell activation are noted in cerebral edema, which is associated with increased permeability of the BBB (Stokum et al., 2016). VEC transcytosis appears to be a critical factor in BBB dysfunction following cerebrovascular stroke (Krueger et al., 2013) and endothelial cell transcytosis is proposed to cause loss of integrity of the barrier with consequential damage to the surrounding structures.

In the present study, there were also abnormalities noted in the pericytes with edematous changes, and pericyte detachment in specimens from MD. Pericyte-endothelial interactions are known to be a critical to maintain the integrity of the BBB and the basement membrane and endothelial tight junction structure are affected by pericyte interactions (Liu et al., 2012). Pericytes contribute to the BLB function in the stria vascularis via upregulation of the tight junction proteins (Shi, 2010; Dai and Shi, 2011; Neng et al., 2013). In the animal model of diabetic retinopathy, basement membrane thickening precedes blood retina barrier endothelial cell degradation and pericyte migration (Hainsworth et al., 2002), and oxidative stress induces loss of pericyte coverage and vascular instability (Garcia-Quintans et al., 2016). In the present study, migration of the pericyte with detachment of the pericyte processes was noted in MD, but not in normative nor in acoustic neuroma, and the loss of normal pericyte-endothelial cell interaction is likely associated with BLB damage. Normative BLB specimens exhibited pericytes characterized by fingerlike projections encircling the VEC, similar to the cerebrovascular pericytes (Dore-Duffy, 2008).

#### Basement Membrane

In the present study, there was thickening and disorganization of the perivascular basement membrane of the BLB in MD, and not in normative. These findings corroborate our previous work that demonstrated loss of the normal expression of collagen IV in the subepithelial and perivascular basement membranes in vestibular endorgans from MD (Calzada et al., 2012a). An intact and functional basement membrane surrounding the capillary is likely critical to the function of the BLB and abnormalities may contribute to BLB permeability alterations. The subepithelial basement membrane disruption may also be associated with an altered expression of aquaporins, and our group demonstrated that aquaporin 4 is downregulated and aquaporin 6 is upregulated in the vestibular supporting cells in MD (Ishiyama et al., 2010). It is likely that the increased permeability of the BLB is associated with damage to basement membranes, the extracellular matrix and the vestibular stroma.

### Oxidative Stress and Breakdown of the BLB

Several inner ear pathologies including acoustic trauma, sudden hearing loss, presbycusis and MD are hypothesized to involve dysfunction of the microvascular circulation (Shi, 2011, 2016). Oxidative stress and ROS have been proposed as mediators for microvascular damage in systemic disorders (Beckman and Koppenol, 1996) and in sensorineural hearing loss, MD, and endolymphatic hydrops (Labbe et al., 2005; Capaccio et al., 2011; Teranishi et al., 2012). Ischemic damage to the cochlea is associated with increased expression of iNOS and NO metabolites (Shi and Nuttall, 2003). iNOS is associated with the production of large amounts of NO, which in the presence of ROS, can form peroxynitrite which mediates the nitrosylation of tyrosine. The presence of elevated levels of 3-nitrotyrosine, a marker for protein tyrosine nitration, has been implicated in the pathophysiology of diverse human diseases: Alzheimer’s disease, cystic fibrosis, myocardial malfunction, lupus nephritis, cerebrovascular strokes, and diabetic retinopathy (Duncan, 2003). The presence of the nitro-group in a tyrosine residue is damaging to cell membranes and can also incite an immunological and inflammatory response (Ye et al., 1996). An increased expression of iNOS and nitrotyrosine has been noted in cerebral demyelinating lesions from the autoimmune neurological disease, multiple sclerosis (Liu et al., 2001).

In our present study, we demonstrated a significant increase in the expression of iNOS in the utricular macula from MD, corroborated with an upregulated expression of mRNA for iNOS. The constitutive eNOS was not more highly expressed in MD patients compared with normative specimens. Under normal conditions, eNOS produces NO at physiological levels, necessary for downstream cellular messengers within the inner ear (Takumida and Anniko, 2004). iNOS, on the other hand, is only detected in the inner ear under pathologic conditions (Takumida and Anniko, 1998a, 2001), and likely can be synthesized by any of the cells of the vasculature when stimulated by cytokines. It is hypothesized that the level of NO production defines its role in the cells as protective or destructive (Albrecht et al., 2003). When NO levels are elevated along with ROS, mitochondria function is blocked and cellular apoptosis may be induced (Clementi et al., 1998; Brüne et al., 1999).

Upregulation of iNOS has been described in several conditions of the inner ear including hydrops in the animal model (Takumida and Anniko, 1998b, 2001; Hess et al., 1999a,b; Shi and Nuttall, 2003). Intratympanic LPS in animal models was associated with an increase in iNOS expression, and the damage could be limited by an infusion of a competitive inhibitor of iNOS (Watanabe et al., 2000). eNOS upregulation is also seen in acoustic trauma or gentamicin injection (Heinrich et al., 2005, 2006), and eNOS can exhibit uncoupling which is associated with ROS (Vasquez-Vivar et al., 1998; Albrecht et al., 2003; Förstermann and Li, 2011; Li and Förstermann, 2013). In the setting of upregulated iNOS, high levels of NO, and nitrotyrosine, and BLB dysfunction as noted in the present study of MD vestibular endorgan, the presence of eNOS may be contributing to damage as well.

The presence of iNOS in the capillaries of the BLB, and nitrotyrosine in the BLB and the stroma, coupled with the histopathological findings of damage to the endothelial cells and the microvasculature, is strongly suggestive that oxidative stress mediates the damage to the BLB in MD leading to a cascade of events culminating in damage within the vestibular endorgan.

**FIGURE 9:**
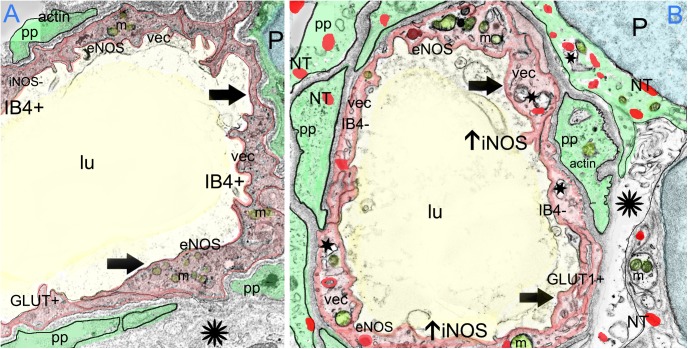
The effect of oxidative stress in the BLB in Meniere’s disease. Meniere’s disease may be associated with a chronic inflammatory oxidative stress response within the microvasculature of the BLB as suggested by upregulation of oxidative stress markers iNOS and the presence of nitrotyrosine. The formation of ROS causes further cellular injury. Nitric oxide may also alter microvascular blood flow and VEC permeability causing loss of the integrity of the BLB through increased endothelial cell transcytosis, pericyte contraction and pericyte migration. VECs may become further damaged by reactive oxygen species and nitration of proteins, followed by increased permeability, allowing an extravasation of fluids and proteins, damaging the extracellular matrix and perivascular basement membranes, and causing associated stromal edema. **(A)** Depicts the normal BLB. **(B)** Depicts the effect of oxidative stress in the BLB in Meniere’s disease.

### Other Models of BLB Damage Due to Induction of Oxidative Stress

Meniere’s is a uniquely human disease, and no animal model produces the symptoms of intermittent vertigo spells with hearing loss. Therefore, it is useful to look into oxidative stress factors in animal models of other otopathologies. In the rodent model of noise-induced hearing loss, TEM analysis reveals similar strial BLB pathology as in our studies: cytoplasmic vacuolization of the endothelial cell, irregular positioning of pericyte processes, and intercellular space between the pericyte and endothelial cell (Shi, 2009). In the diabetic rat model, both iNOS and eNOS were upregulated in the cochlea, indicative of oxidative stress. BLB damage was evidenced by extravasation of Evans blue, associated with hearing loss (Liu et al., 2008). Noise exposure in the mouse knock out for manganese superoxide dismutase, an endogenous antioxidant, causes significantly more outer hair cell damage than normative (Tuerdi et al., 2017), and noise exposure induces nitrotyrosine production within the outer hair cells, associated with apoptosis of the outer hair cells (Han et al., 2013). There may be a genetic susceptibility in patients with MD, with polymorphism in one gene involved in oxidative stress noted in higher expression in a Japanese population study (Teranishi et al., 2012).

There is evidence that strial BLB permeability change in noise exposure is mediated by a decreased expression of occludin, a tight junction associated protein (Shi and Nuttall, 2003; Zhang et al., 2013; Wu et al., 2014). The production of inflammatory mediators from the capillary VECs is hypothesized to trigger a cascade of subsequent events (Trune and Nguyen-Huynh, 2012). Pharmacological interventions targeting tight junction protein expression by pericytes are being developed for downregulation of oxidative stress proteins in stroke and cardiac arrest (Ronaldson and Davis, 2015). Further studies are indicated to evaluate the integrity of and expression of proteins in the tight junctions of the BLB of MD.

Pericytes may mediate a decreased capillary reflow due to oxidative-nitration mediated pericyte constriction (Yemisci et al., 2009). Thus, it is not clear whether the NO production is necessarily only detrimental, and indeed, the upregulation of NO production may be an attempt to vasodilate in response to hypoxia due to vasoconstriction. Further studies are indicated to evaluate for oxidative stress products, immunolocalization on a cellular level, to discover new potential targets for the treatment of intractable MD.

### Summary of Oxidative Stress in the BLB of Meniere’s Utricles

**Figure 9A** depicts the normal BLB, and **Figure 9B** depicts the changes due to oxidative stress in the BLB in MD that have been documented in the present study. We have shown that MD is associated with an upregulation of iNOS expression within the microvasculature of the BLB, along with constitutive expression of eNOS, the combination of which are likely associated with high levels of NO. The endothelial cell of the BLB exhibits nitrotyrosine expression, likely associated with increased microvascular permeability, VEC transcytosis into the subepithelial stromal utricular macula, pericyte migration and endothelial cell damage. The increased permeability of the BLB may be causative of the interstitial edema, stromal vacuolization, and damage to the basement membrane and extracellular matrix that our group has documented in prior studies (McCall et al., 2009; Ishiyama et al., 2017). The nitration of proteins may be also associated with neuroepithelial edema and damage to the sensory neuroepithelium as noted in this study with the presence of nitrotyrosine, within the neuroepithelium and stroma. A similar process may occur in the hearing system to cause hearing loss of MD.

## Conclusion

The cellular and structural changes suggest that the VECs in the microvasculature of the BLB are damaged due to oxidative stress in MD. The VECs express iNOS and nitrotyrosine, exhibit degeneration, loss of organelles, loss of IB4 expression, and excessive transcytosis. Pericytes exhibit vacuolization and migration with loss of the encircling fingerlike projections. These degenerative changes of the BLB may be causative of the degeneration of the perivascular basement membrane and the extracellular matrix, as has been documented in our prior studies. These findings confirm with histopathology the MRI evidence for a loss of integrity of the BLB in MD and are strongly suggestive that oxidative stress plays a critical role in the pathophysiology of MD. Quantitative stereological studies at the transmission electron microscopy level of the microvasculature are warranted to further understand the cellular and molecular biology of the BLB in normative and MD and such studies are integral for the development of therapeutic agents which may block the BLB inflammatory response.

## Author Contributions

GI development of the theory, designed the project, interpretation of histopathology and all studies, mentor and oversee the studies, interpretation of clinical data, experimental design, approve and work on final manuscript. JW performed immunofluorescence staining, interpretation of studies, writing first draft, approve and work on final manuscript. IL development of the theory, designed the project, participated and mentored in all aspects of the experiments, prepare the figures, approve and work on the final manuscript. LB-P performed gene expression array experiments and interpretation of its results. AI design of the project, collection of specimens, interpretation of clinical data, experimental design, approve and work on the final manuscript.

## Conflict of Interest Statement

The authors declare that the research was conducted in the absence of any commercial or financial relationships that could be construed as a potential conflict of interest. The handling Editor declared a shared affiliation, though she has no past or active collaboration with the authors prior to the editorial assignment.

## Footnotes

1 https://imagej.nih.gov/ij/download.html

## Abbreviations

Alpha SMA: alpha smooth muscle actin
BBB: blood–brain barrier
BLB: blood labyrinthine barrier
eNOS: endothelial nitric oxide synthase
GLUT 1: glucose transporter 1
IB4: isolectin B4
iNOS: inducible nitric oxide synthase
MD: Meniere’s disease
NTIF: nitrotyrosine immunofluorescence
ROS: reactive oxygen species
VEC: vascular endothelial cell
